# CW ESR spectroscopy and protein spin labeling in membrane biology

**DOI:** 10.1088/1478-3975/ae2db1

**Published:** 2025-12-30

**Authors:** Olamide Ishola, Adeyemi Ogunbowale, Emma Abdul-Rahman, Katie Starr, Pengyu Zhu, Peter P Borbat, Elka R Georgieva

**Affiliations:** 1 Department of Chemistry and Biochemistry, Texas Tech University, Lubbock, TX 79409, United States of America; 2 Department of Chemistry and Chemical Biology and ACERT, Cornell University, Ithaca, NY 14853, United States of America

**Keywords:** membrane proteins, membrane protein conformational dynamics, protein spin labeling, continuous wave electron spin resonance (CW ESR), study of membrane protein by CW ESR

## Abstract

Biological membranes define cellular and organelle boundaries, and perform vital functions, providing transport, recognition, signaling, and interaction with other cells. These membranes are majorly composed of lipid bilayers and membrane proteins. Membrane proteins perform most membrane functions. Based on their localization, they are classified as integral and peripheral proteins. In this overview, we provide basic information about membrane proteins structure, conformational dynamics, and functions, and outline the methodologies used to produce highly-pure functional membrane proteins for *in vitro* biophysical characterizations based on selected examples. To this end, expression of membrane proteins in a host, their extraction, purification and reconstitution in model lipid bilayers are described. Further, biophysical approaches play key role in elucidation of the structure and function of membrane proteins. Our focus here is on the technique of continuous wave electron paramagnetic/spin resonance (CW ESR) spectroscopy applied to spin-labeled membrane proteins. We describe the basic principles of membrane proteins labeling with nitroxide spin labels (paramagnetic tags) and how the CW ESR can be successfully used in elucidating the conformational dynamics of such proteins. We describe the basic principles of the CW ESR technique. The capability of this technique to characterize physiologically relevant conformational dynamics of proteins is demonstrated using two examples of CW ESR studies on spin-labeled human Tau and influenza A M2 proteins. The method is highly suitable to study physiological structure-function relationships of a broad range of proteins, and to explain the malfunctional states of proteins linked to diseases. This review is directed to the broader biophysical community with interest in molecular biophysics of biological membranes.

## 1. Biological membranes and membrane proteins

Membrane proteins fulfill important biological functions. They may be localized either at the membrane surface or span the membrane, and certain proteins possess the capability to transition between a soluble form and a membrane-bound state. Biological membranes are generally bilayers of lipid molecules, which provide the environment for housing membrane proteins [1].

**Figure 1.**
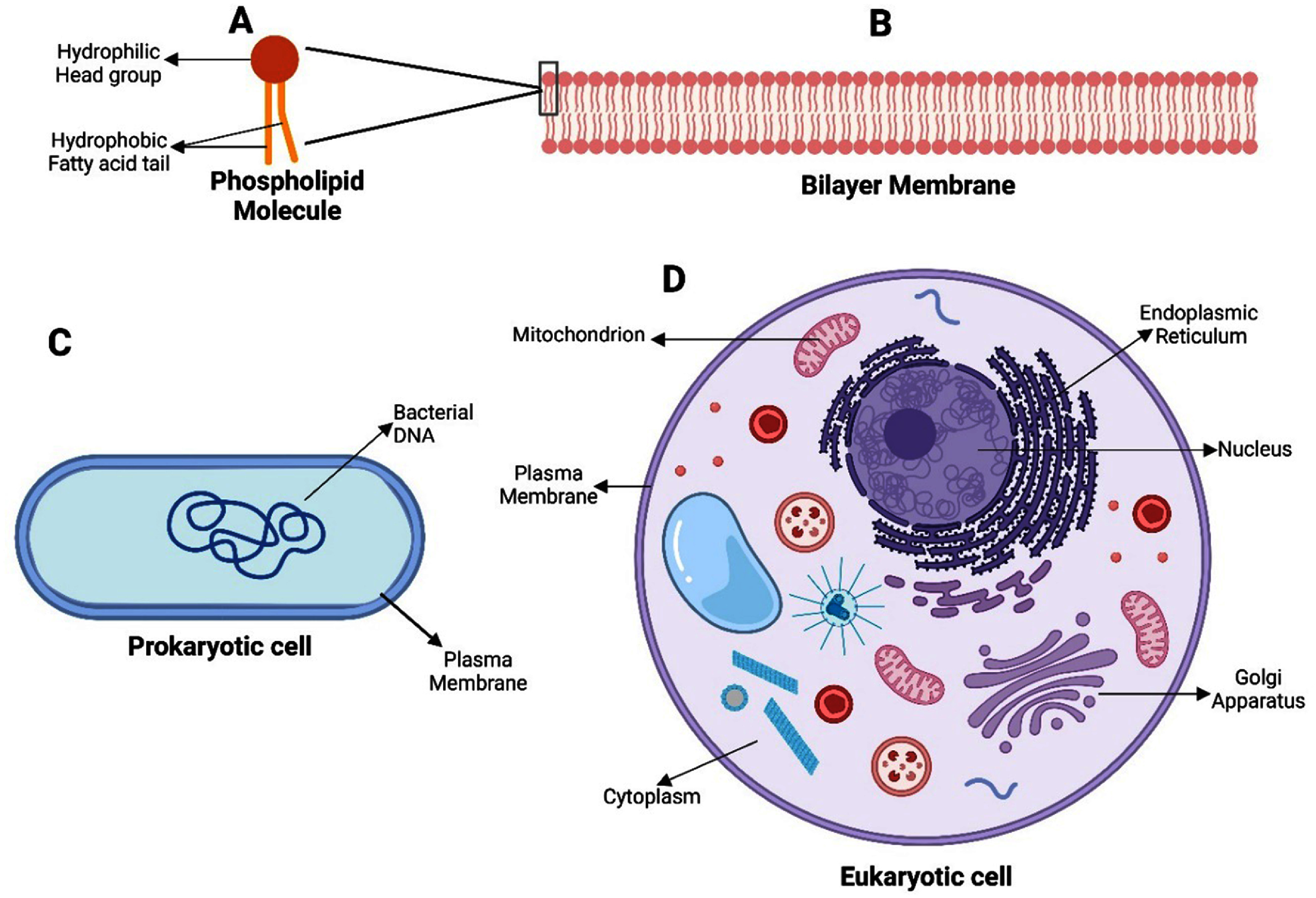
Biological membranes are composed of lipid bilayers and membrane proteins. (A) Schematic representation of a lipid (phospholipid) molecule containing a hydrophilic headgroup and hydrophobic hydrocarbon tails (typically 2 tails). (B) In biomembranes, the lipid molecules are arranged into bilayer structure, exposing the hydrophilic headgroups to aqueous environment and shielding the hydrophobic core of the membrane. The two leaflets generally have different lipid compositions. (C) Prokaryotic cells have only one membrane, which surrounds the cell and defines the cell interior. This structure is known as the plasma membrane. In addition to plasma membrane, some prokaryotes (bacteria) have an outer bacterial wall (not shown). (D) Eukaryotic cells possess, in addition to a plasma membrane, several distinct intracellular compartments; for example, nucleus, Golgi apparatus, endoplasmic reticulum, mitochondria, etc, called organelles. Every organelle is surrounded by its individual membrane. Prokaryotic cell ‘organelles’ such as flagella or receptor arrays are not encased in special membranes.

**Figure A1.**
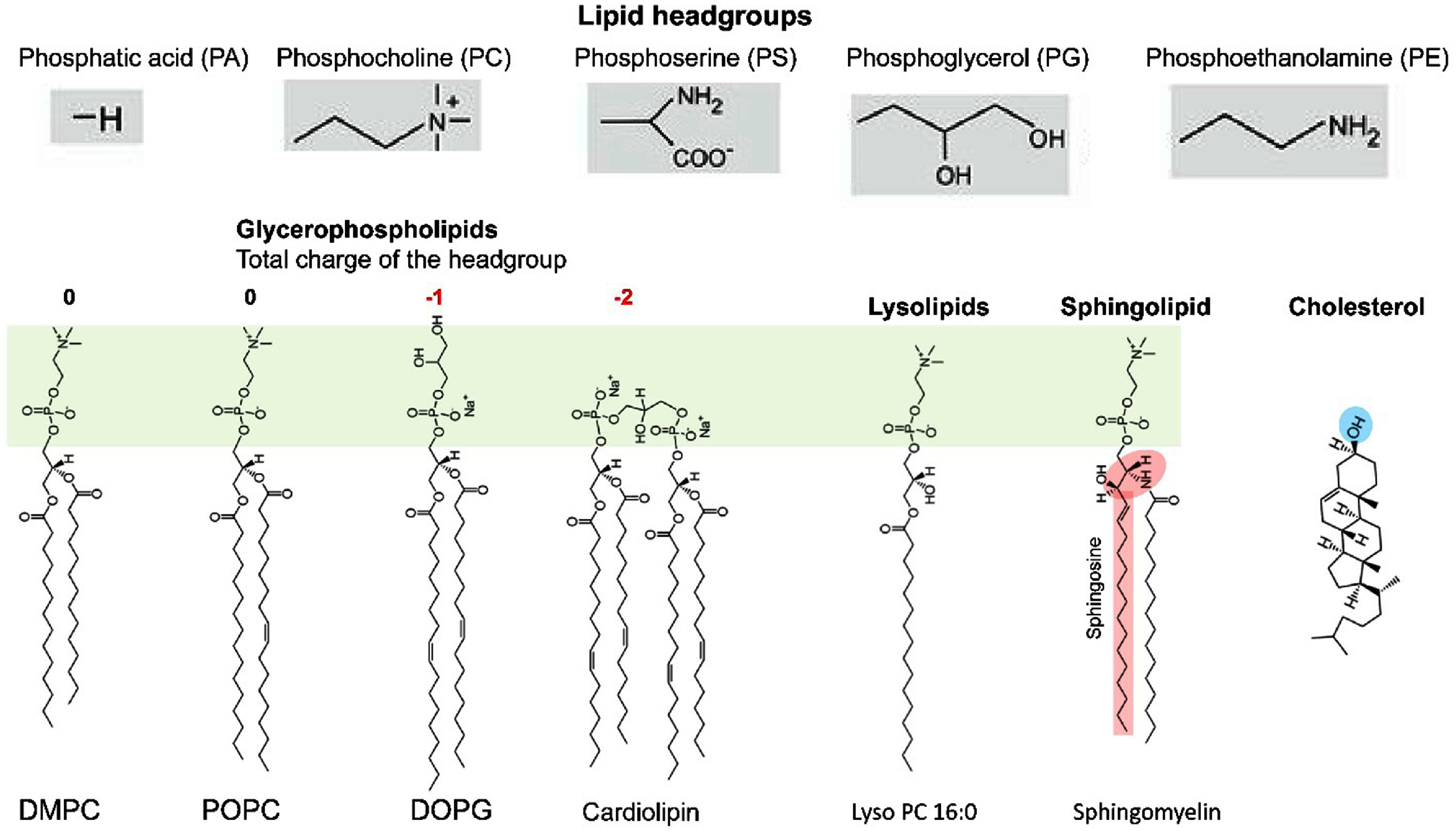
(Top panel) some of the most common headgroups of lipids found in biological membranes are shown: these are the headgroups containing just hydrogen atom bound to the phosphate (forming phosphatidic acid headgroup, PA) or either choline, serine, glycerol, and ethanolamine bound to the phosphate group via a phosphodiester bond, resulting in, respectively, phosphocholine (PC), phosphoserine (PS), phosphoglycerol (PG), and phosphoethanolamine (PE) headgroups. These headgroups are highlighted in green in the bottom panel where the structures of the whole lipid molecules are shown.

Biological membranes are composed mainly of lipids and membrane proteins [1]. Phospholipids are the most abundant lipids in these membranes, containing a hydrophilic headgroup (can interact with water molecules) covalently linked to two long hydrophobic (not being able to interact with water molecules) hydrocarbon tails (figure 1(A)). In the membrane, the lipid molecules are assembled into a bilayer structure made of two leaflets with the hydrophilic headgroups facing the aqueous environment and hydrophobic tails protrude inside the bilayer and interacting with each other to provide stability of the membrane [1, 2] (figure 1(B)). Each cell in unicellular and multicellular organisms is surrounded by a plasma membrane (figures 1(C) and (D)). Further, the eukaryotic cells have intracellular organelles, each having their special membrane defining the organelle inner and outer (cytosolic) space (figure 1(D)). The lipids found in prokaryotic plasma membrane and in the eukaryotic plasma and organelle membranes differ significantly mostly in the composition of their headgroups. Thus, the main phospholipids in bacterial membrane are phosphatidylethanolamine (PE), phosphatidylcholine (PC), phosphatidylglycerol (PG), and cardiolipin (CL) [3]. PC is a major lipid in the eukaryotic membranes, followed by PE, as well as lower proportions of anionic lipids phosphatidylserines (PS) and phosphatidylinositols; however, unlike the Golgi and endoplasmic reticulum membranes, which contain just about 5 mol% cholesterol (Chol), the PM contains up to 50 mol% Chol and includes sphingomyelin [4–7]. The inner mitochondrial membrane has a high concentration of CL [7]. Various lipid headgroups and structures are briefly illustrated in the Appendix figure A1.

**Figure 2.**
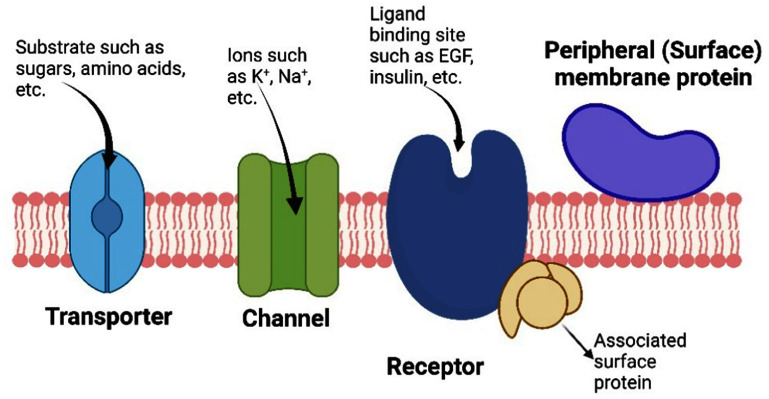
Membrane proteins reside in or at the membrane lipid bilayer. Some proteins, called integral membrane proteins, span the membrane and transport solutes and ions across the membrane, i.e. they are either transporters or channels—shown on the left, but can also transduce signals from outside to inside the cell (receptors). Other proteins associate with the surface of the membrane by interaction with either lipid headgroups or soluble regions of membrane proteins. These proteins are referred to as peripheral or surface membrane proteins.

Membrane proteins reside in or associate with the respective plasma and organelle lipid bilayers completing the structure of biological membranes (figure 2) [8, 9]. These proteins fulfill important physiological functions depending on their localization. The integral membrane proteins fully or partially traverse the membrane (figure 2, left). The proteins traversing the plasma membrane completely (transmembrane proteins) can transduce signals across the membrane, thereby providing communication between the cellular interior and exterior—they are called receptors; other transmembrane proteins can transport across the membranes various solutes, such as ions or small molecular (e.g. sugars, amino acids, etc)—they are called ion channels and transporters, respectively [1]. Other types of proteins associate with membrane periphery (surface membrane proteins) by binding to the membrane lipid molecules or regions of integral membrane proteins, which protrude outside of the lipid bilayer (figure 2, right). They also have important physiological roles, such as binding to and further communicating the signal transduced by the receptor or they may be a part of a metabolic pathways [10, 11].

It is important to point out that, in addition to the organism’s native membrane proteins encoded by its DNA, viruses also contribute to the membrane proteome—the full range of membrane proteins—of infected cells. Viral membrane proteins are produced in host cells and often reside within cellular membranes. These viral proteins can function as ion channels, interact with host proteins, or trigger virus uncoating and assembly, thereby modifying processes in the infected cell and aiding the virus [12–14].

**Figure 3.**
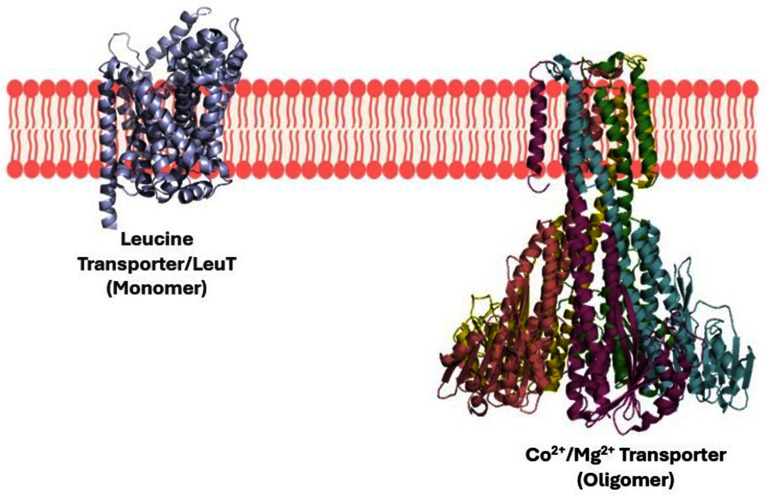
Membrane proteins can be either monomers or oligomers. Monomeric transmembrane proteins are illustrated with LeuT transporter (PDB# 3TU0) (left) and homo-oligomeric transmembrane proteins are illustrated with Co^2+^/Mg^2+^ transporter CorA (PDB# 4I0U) (right)—a pentamer with monomers shown in distinct colors.

To fulfill their function, the membrane proteins typically fold into distinct 3-dimensional structures [1]. While some membrane proteins function as monomers with no need to interact with other proteins to form homo- or hetero-complexes (oligomers), others do assemble into oligomers comprising more than one monomer (also called subunit) of the same or a different type in the protein complex (figure 3). In eukaryotes the individual subunits can be linked into a single polypeptide, thereby greatly increasing their catalytic or transport efficiencies and reducing cell loading with dysfunctional monomers.

**Figure 4.**
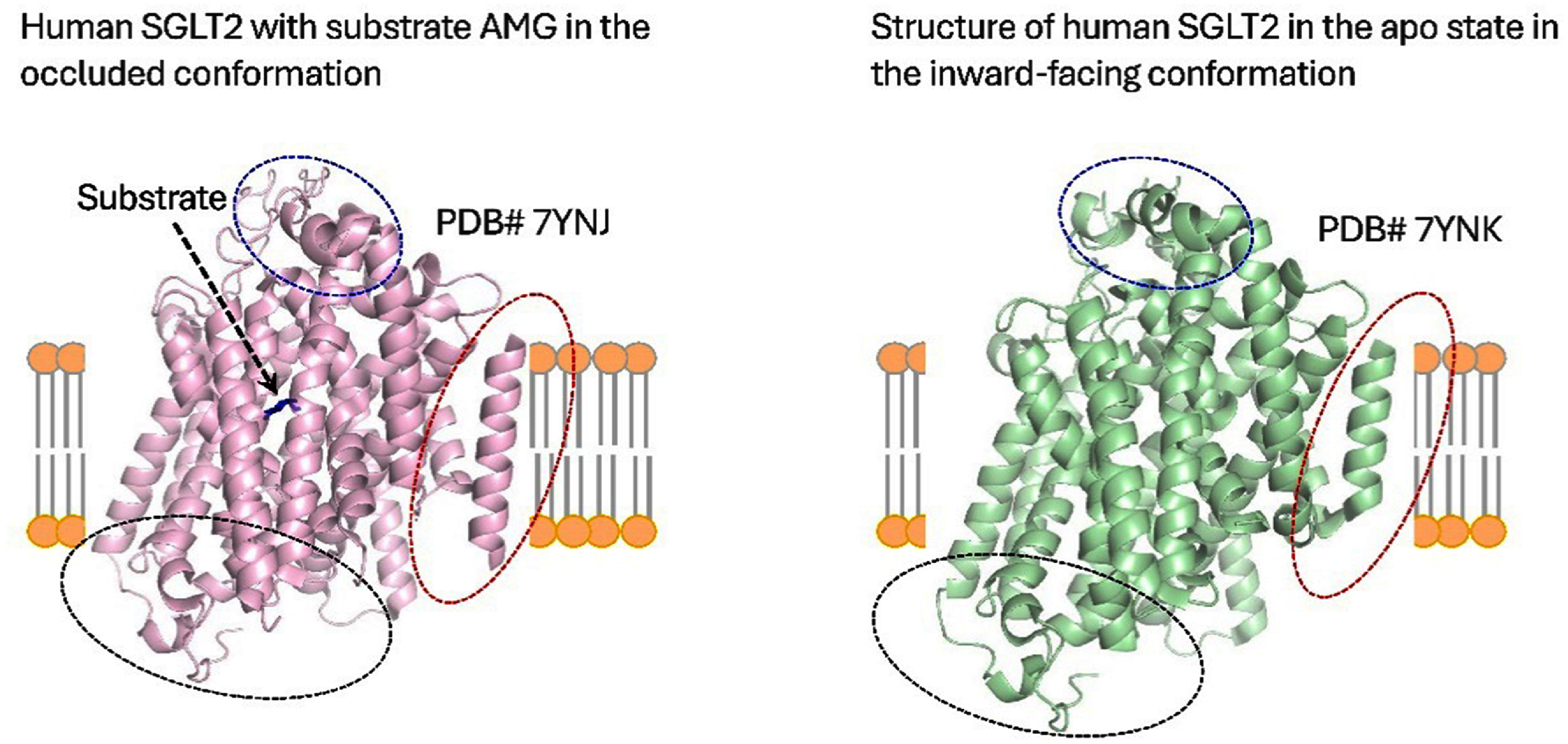
The 3-dimensional structures of SGLT2 transporter in two distinct functional conformations. The occluded conformation with a molecule of substrate methyl alpha-*d*-galactopyranoside (AMG) bound in an occluded substrate-binding site (at the center of the protein) is shown on the left. The substrate-free (apo) protein in inward facing conformation is shown on the right (the protein is open to the cytoplasm). Subtle but distinct conformational rearrangements take place during the transport cycle. Protein regions, which undergo structural rearrangements to switch conformations upon substrate translocation, are encircled in red, blue and black. The protein data bank (PDB) identification numbers of each of the structures are shown as well.

Importantly, membrane proteins do not exist as just static 3-dimensional structures. They are dynamic and transition between several distinct, typically close in organization conformational states. They can be viewed as ‘nanomachinery’ humming collectively in the organism like in a factory. Protein dynamics and structural plasticity are key to their function. For example, the human SGLT2 transporter undergoes conformational changes upon binding and releasing the substrate [15] (figure 4). The SGLT2 is a glucose transporter in the kidney, which is linked to type 2 diabetes; consequently, it is a target for inhibition [16, 17]. Therefore, knowing its structure-function relationship is important.

## 2. Production, cysteine mutagenesis and spin labeling of membrane proteins

### 2.1. Production of membrane proteins and their reconstitution in lipid bilayers

**Figure 5.**
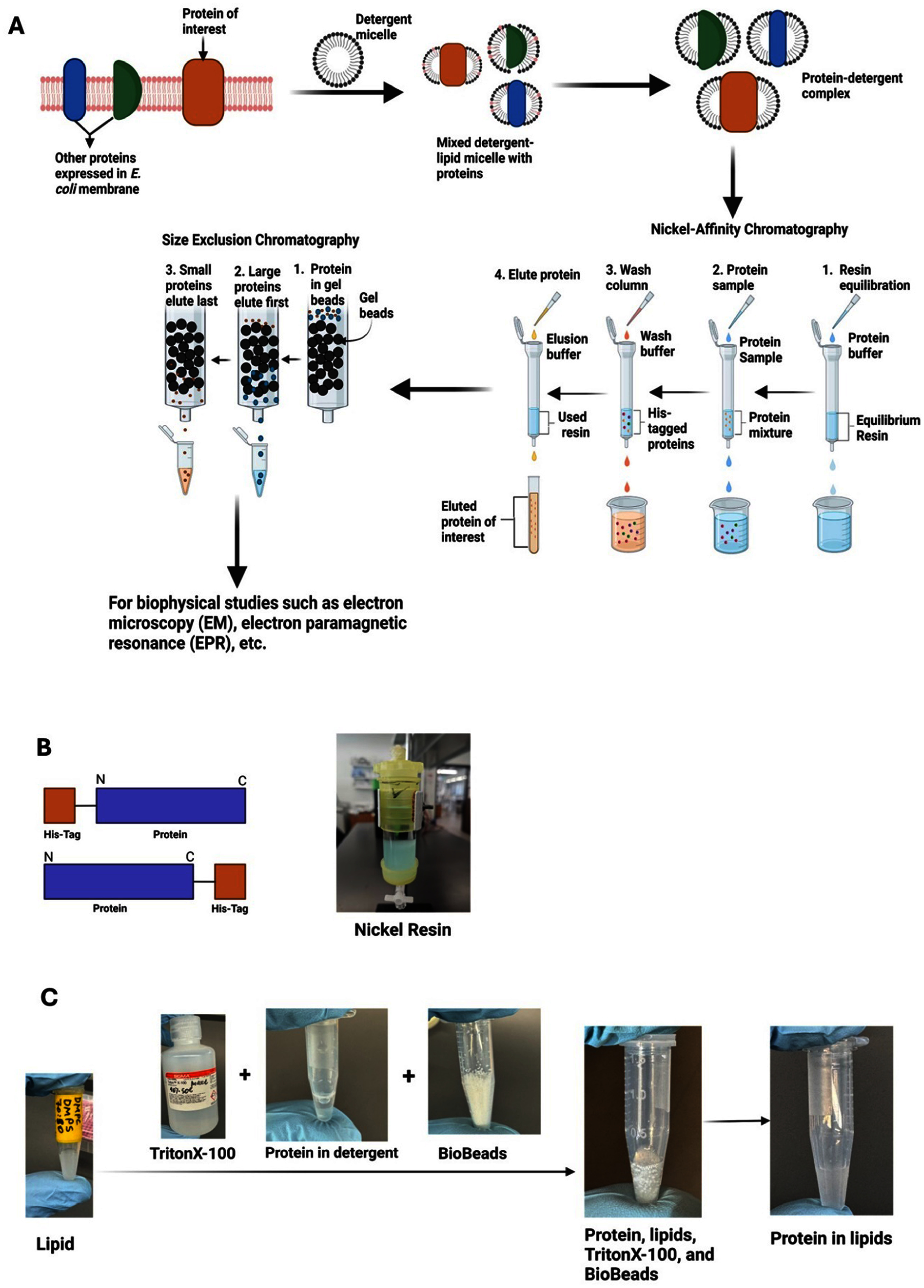
The steps used for *in vitro* extraction, purification, and membrane reconstitution of membrane proteins. (A) The extraction of membrane proteins from the host membrane using detergent micelles is illustrated. The protein of interest, heterologously expressed in the host membrane, is in orange. It is separated from the host’s native membrane proteins in the process of protein purification. Most of the purification protocols include Ni-affinity chromatography followed by size-exclusion chromatography. (B) Schematic representation of an engineered protein construct having the His-tag containing typically 6–10 histidine residues at either the N- or C-terminus (left) and the nickel ions immobilized on beads (Ni-resin) loaded onto a gravity column (right) are shown. (C) The reconstitution process of purified protein into lipid membrane mimetics is shown: the protein in detergent is combined with lipid and mild detergent (Triton X-100) followed by detergent removal using BioBeads^TM^ SM-2 resin (hydrophobic microporous polymeric beads, which bind only the detergent) to form protein-lipid complexes (liposomes).

For their *in vitro* studies, membrane proteins need to be purified and then reconstituted in lipid membrane mimetics. Typically, the proteins are expressed in large quantities in a host, which can be the bacterium *Escherichia coli* (*E. coli*), yeast, mammalian cells, etc [18, 19]. Surface membrane proteins can be expressed as soluble proteins or detached from the host membrane surface by using, for example, high salt concentration (if the surface membrane protein interacts with the membrane or other membrane protein via electrostatic interactions). Transmembrane proteins can also be expressed in a soluble form outside the membrane; however, these are typically engineered chimera constructs of the protein of interest fused to a highly soluble or amphipathic (having dual nature, i.e. hydrophobic and hydrophilic) fusion partner [20, 21]. Most commonly, the transmembrane proteins are expressed in the host membrane and then extracted from these membranes using high concentration of detergent (figure 5(A)) [22]. Thereafter, a membrane protein of interest stabilized in buffered solution containing detergent is purified, most frequently, by using nickel (Ni)-affinity chromatography followed by size-exclusion chromatography (figure 5(A)). For Ni-affinity chromatography, the protein is modified to include a poly-histidine tag (His-tag) at either the N- or C-terminus (figure 5(B)) [23]. The detergent-purified protein (purity from 80% to nearly 100%, depending on the experimental requirements) is then reconstituted in lipid bilayers mimicking the native membranes. To this end, the lipid mixtures prepared in buffered aqueous solutions as lipid suspensions are solubilized with Triton X-100 (a mild nonionic detergent) and then mixed with detergent-purified proteins at desired protein-to-lipid molar ratios. The detergents are subsequently removed using BioBeads SM-2 resin (figure 5(C)) [24, 25].

For continuous-wave electron spin resonance (ESR) (CW ESR) studies, which are of interest here, the membrane proteins need to be highly pure (i.e. they should be purified to as close to 100% as possible or reasonable) and are typically spin-labeled in detergent-solubilized form before their reconstitution into lipid membranes.

Alternatively, if the membrane protein is of a small size, or when only the membrane-interacting region is studied, the protein can be synthesized (commercially or in the lab), solubilized in buffered detergent solution, spin-labeled, and finally reconstituted in lipid membranes. For example, such approach was used to study the S^21^68 and influenza A M2 proteins [25–27].

### 2.2. Introducing cysteine residues at desired positions in proteins and nitroxide spin labeling

**Figure 6.**
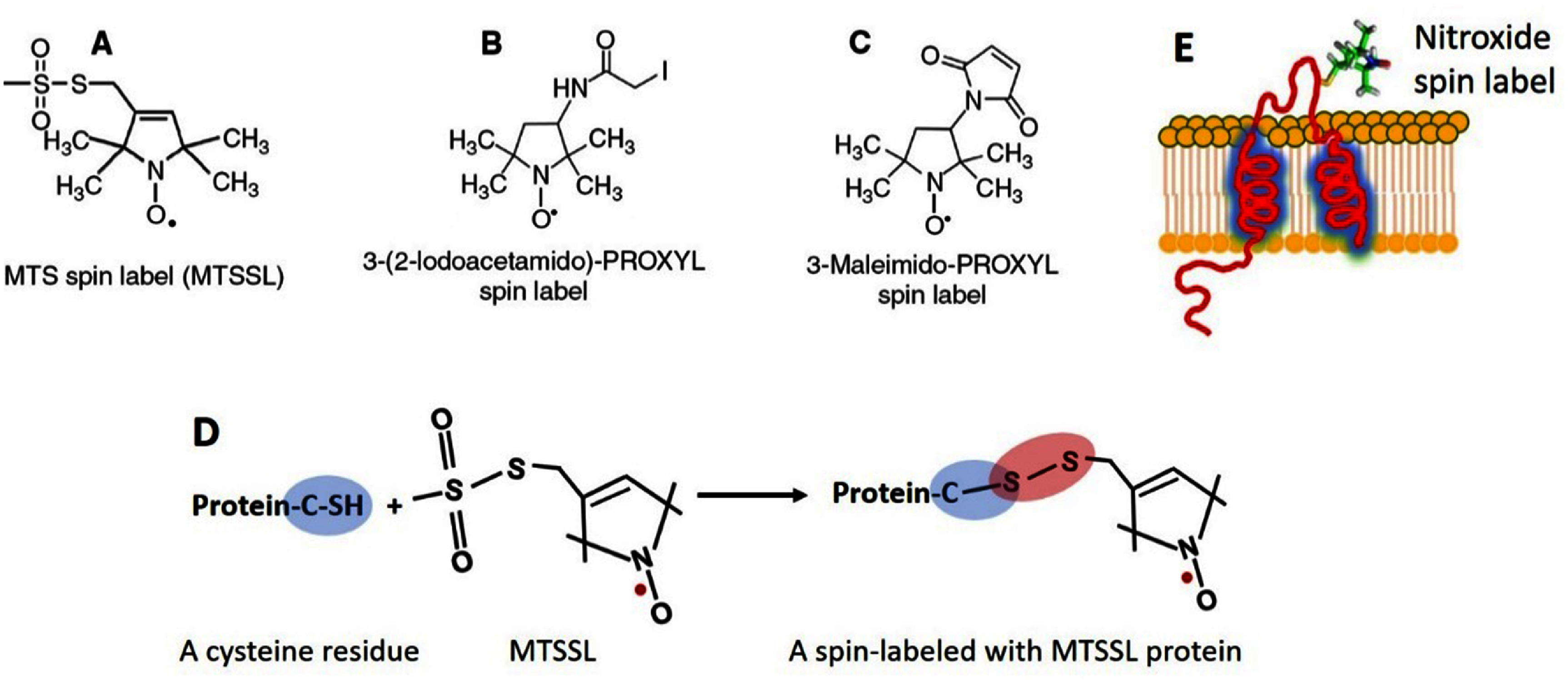
The most widely used nitroxide spin labels: the MTS spin label (MTSL or MTSSL), 3-(2-iodocetamido)-PROXYL spin label and 3-maleimido-PROXYL spin label are shown in (A), (B) and (C), respectively. (D) Schematic presentation of the reaction of cysteine residue in a protein with MTSL resulting in covalent attachment of MTSL. (E) Schematic presentation of a membrane protein spin-labeled with MTSL. The MTSL 3-dimensional structure was produced using the molecular modeling (MMM 2017.2) software [33], which is used to generate an ensemble of the spin-label side-chain conformations (rotamers) at the labeled site. The size of MTSL (and of several other nitroxide labels) is comparable to that of an amino acid of average size, which is rather small and almost non-perturbing to the protein.

**Figure 7.**
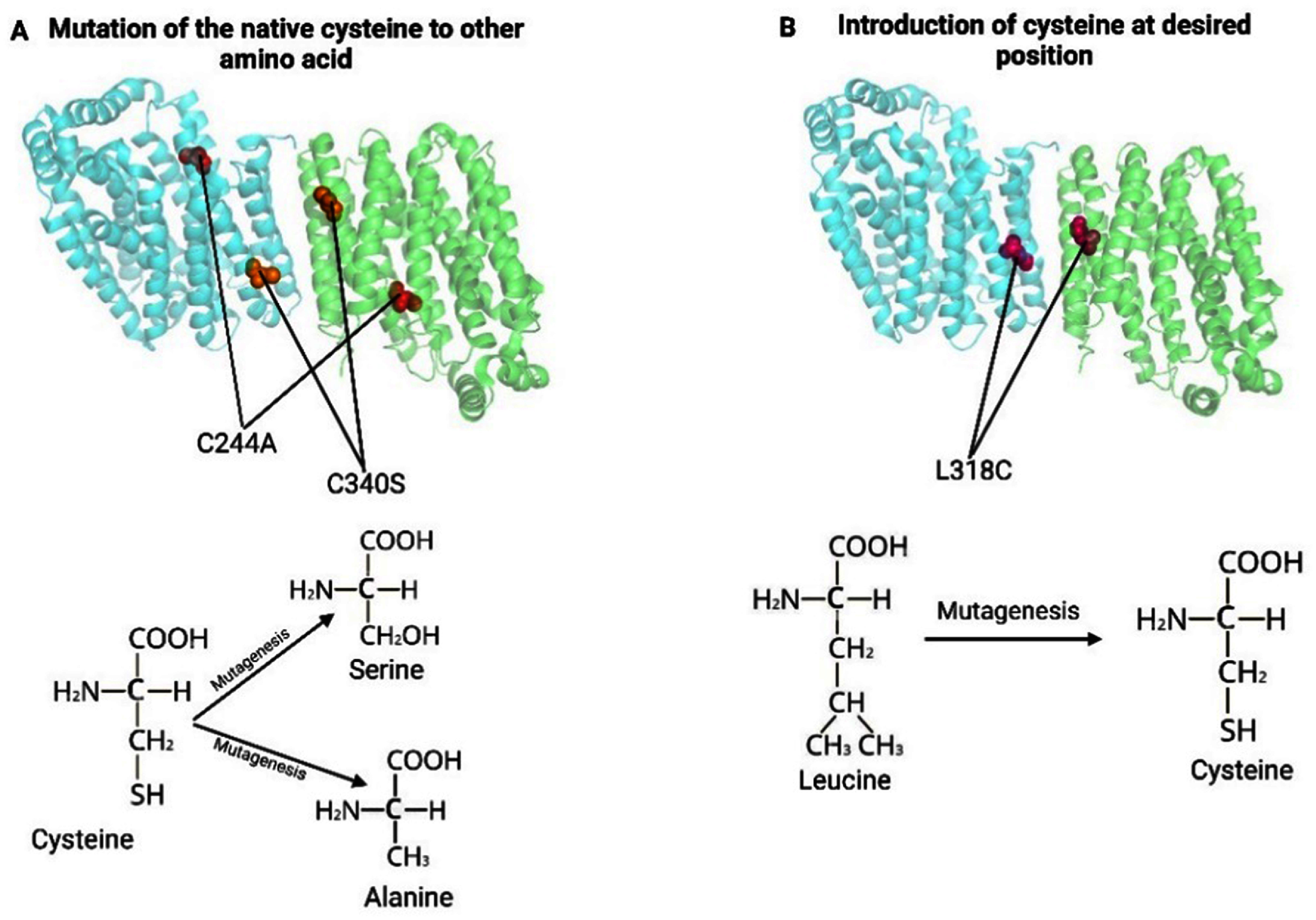
Introducing cysteine residues at desired position in a protein: site-directed mutagenesis is used to mutate out the native cysteine residues (e.g. substitute the cysteines with alanine or serine) and to selectively introduce cysteine mutations by substituting native residue(s) with cysteine(s). The principle is demonstrated using the structure of the homodimeric glucose transporter from *Staphylococcus epidermidis* (PDB# 4LDS).

To study membrane proteins, and proteins in general, using ESR (also known as electron paramagnetic resonance, or EPR) spectroscopy, these proteins must have paramagnetic centers that have unpaired electrons. However, only few proteins have such a property—these are proteins containing endogenous radicals (e.g. based on FAD or tyrosine radical cofactors) or coordinating transition metal ions. Therefore, the first step in studies of conformational dynamics of membrane proteins using CW ESR usually includes incorporation of covalently bound paramagnetic reporter groups referred to as spin labels. Besides, binding of spin-labeled substrate, inhibitor, or a recognition peptide was also used [28, 29]. Currently, the most widely used spin labels are based on nitroxide radicals and have either methanethiosulfonate (MTS), maleimide, or iodoacetamide functional groups, which can react with the sulfhydryl (thiol, -SH) group of cysteine amino acid residues in proteins’ polypeptide chains (figure 6). To this end, cysteine residues need to be introduced at selected locations in the protein polypeptide chains via site-directed mutagenesis. In many cases all or most of the native cysteine residues in the protein are mutated out to other amino acids, often alanine or serine; and then the cysteine mutations at the desired positions are introduced (figure 7). Typically, the membrane protein of interest should contain just a single cysteine residue for spin labeling and CW ESR studies because if more than one cysteine residues are spin-labeled they will produce overlapping CW ESR spectra of spin labels linked to residues having different mobility. Further information on proteins (membrane proteins) spin labeling can be found in other works focusing on this area [30–32].

## 3. The CW ESR technique and instrumentation

There is a variety of biophysical methods employed to study lipid bilayers and membrane proteins, including protein-associated lipids, fatty acids, and fats. In this article, we focus on CW ESR spectroscopy, which is one of the magnetic resonance methods used in biophysics. Magnetic resonance in general is based on detection of resonant absorption of electromagnetic radiation by the tiny magnetic moments of molecules and atoms that are nuclear spins in NMR and electron spins in ESR.

### 3.1. Basic principles of CW ESR

ESR requires unpaired electron spins for detection, such as those of paramagnetic ions, free radical species, etc. Due to the complexity of the method, here we provide only the very basic description of it. The electrons of a chemical bond in organic compounds and biomolecules are paired in atomic or molecular orbitals where they reside, giving zero total electron spin. The unpaired electrons in biological systems are typically those of radical species or transition metal ions, for example those native or else purposely introduced into proteins. By studying these paramagnetic centers in proteins using ESR, one can obtain valuable information regarding protein structure, conformational dynamics, and function.

CW ESR spectroscopy detects the resonant absorption of CW electromagnetic radiation in paramagnetic samples containing unpaired electron spins. To enable this absorption, RF radiation, usually in microwave (MW) range, is applied to the sample to induce the transitions between the energy levels created by the electron spin Zeeman interaction with the polarizing static magnetic field and by the hyperfine (hf) interaction of the electron with the spins of surrounding nuclei.

**Figure 8.**
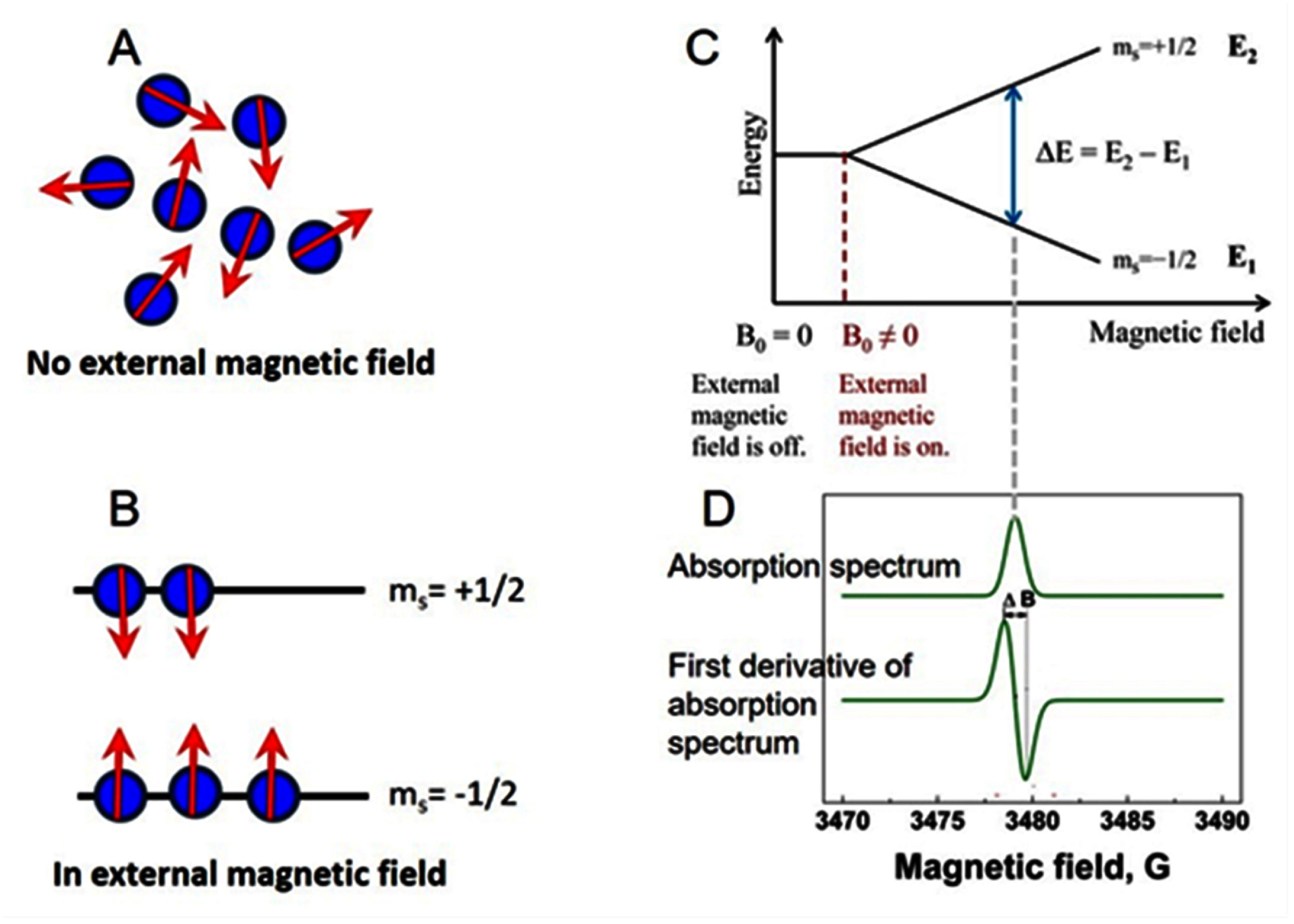
The basic principles of CW ESR spectroscopy: (A) in a paramagnetic medium, molecules bearing unpaired electron spins **S** have the same energy, their magnetic moments ***µ***_e_ = *g*_e_*β***S** are randomly-oriented and the sample magnetization is zero. (B) In the external magnetic field **B**, the electron spin Zeeman interaction ***µ***_e_**B** of the electron spin magnetic moment with the field **B** results in two energy levels *E*_1,2_ = ± *g*_e_*βB*/2 for electron spin magnetic quantum numbers *m*= ± 1/2, respectively. (In the above text, *β* is the Bohr’s magneton, *g*_e_ is the electron spin *g*-factor). The level splitting Δ*E* = *E*_2_–*E*_1_ is thus *g*_e_*βB*. The level populations are slightly different due to the Boltzmann factor exp (–Δ*E*/*k*_B_*T*) with the thermal level population equilibrium mediated by relaxation, so the sample becomes magnetized, having macroscopic magnetic moment parallel to **B**. (C) The MW quanta with frequency *ν* corresponding to the splitting Δ*E* as Δ*E* = *hν, h* is the Planck constant, cause transitions between the two levels. (D) The absorption line shape is recorded in CW ESR as the first derivative by using an amplitude modulation of *B* in audio range and phase-sensitive detection of the ESR signal.

In a dilute paramagnetic sample unpaired electron spins **S**, depicted in figure 8(A) as red arrows, are far enough from each other and can be considered non-interacting and are randomly oriented, i.e. the energy of the electron having magnetic quantum number *m* of –1/2 and +1/2 is the same in the absence of the polarizing external magnetic field, hence the sample magnetic moment is zero.

When the external magnetic field **B** is applied, the electron spin energy is split into two levels (figure 8(B)) by the electron spin Zeeman interaction, *β***B g**_e_ **S.** The electrons with *m =*–1/2 occupy the low energy level and those with *m* = + 1/2 are at the high energy level separated by Δ*E* = *g*_e_*βB*. At thermal equilibrium, the Boltzmann factor exp(–Δ*E* /*k*_B_*T*) results in level population difference (figure 8(C)) giving rise to sample magnetization **M**= *χ***B**. When a paramagnetic sample in the external magnetic field -**B** is irradiated with MWs, resonant MW radiation causes transitions between the levels; this tends to equalize their populations leading to zero net absorption, i.e. complete saturation. The spin relaxation transitions between the two levels in turn tend to restore the thermal equilibrium, so that the combined effects of MW radiation and relaxation results in ESR absorption that depends on MW power and relaxation rates.

This absorption of MW power is recorded using CW ESR spectrometers. An ESR spectrum is a superposition of lines of all possible transitions and usually is much broader than underlying (homogeneous) linewidths for every transition. The ESR spectrum is usually recorded by sweeping ***B*** while keeping MW frequency fixed due to insurmountable difficulties to perform this by sweeping MW frequency. Because of the very low level of power absorbed in a typical ESR sample, the CW ESR instruments as a rule use magnetic field modulation to generate ESR modulation sidebands of ESR absorption signal. The sidebands are away from the high level of the MW source low frequency noise. Consequently, the absorption is synchronously detected as the first derivative of the spectrum, as shown in figure 8(D) (lower line) and, in optimal conditions, the larger is the modulation amplitude and MW power and sharper the line the stronger is the signal. But the maximum MW power is often limited by the saturation of the spectrum and field modulation amplitude is limited by the spectral linewidth. And, of course, both cannot be too large due to technical limitations or to avoid thermal or other unwanted effects. More detailed description of the CW ESR method can be found in [34] or just by reading the Xenon user’s manual initial insights can be gained.

### 3.2. CW ESR on nitroxide spin-labeled proteins

**Figure 9.**
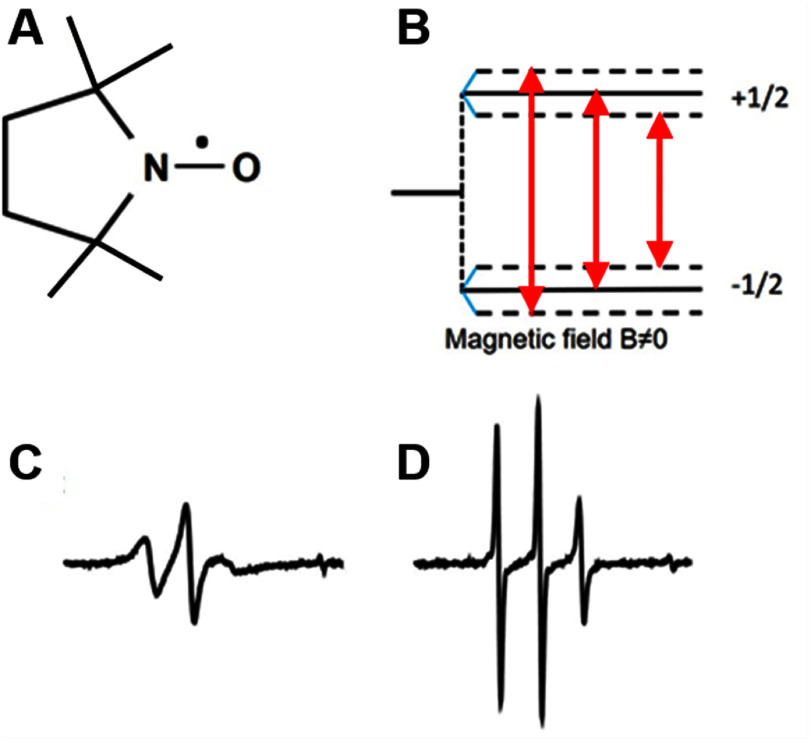
The CW ESR spectrum of a nitroxide radical (spin label): (A) A nitroxide moiety with NO group containing unpaired electron spin is shown. (B) The hyperfine interaction with ^14^N nuclei splits each Zeeman level into three hf sublevels, giving rise to 3 allowed transitions shown in red. (C) Typical nitroxide spectrum in a highly viscous solution or else with the motion restricted by the attachment to a protein is shown. (D) The spectrum of a nitroxide spin label rapidly tumbling in a low-viscosity solvent or attached to highly dynamic protein region is shown.

In the case of spin label, the electron spin density of the nitroxide radical is partially delocalized on the nitrogen atom (figure 9(A)), which has a nuclear spin of 1.

The magnetic moments of electron spin **S** and of the nitrogen nucleus spin **I** interact by the isotropic Fermi contact interaction and anisotropic dipole-dipole interaction resulting in orientation-dependent hf splitting of the energy levels. hf interaction is **I.A.S**, where **A** is the hf interaction tensor which in a given orientation of the NO bond splits the energy levels as *E*(*m, M*) = *m* (*βg*_e_*B*+ *aM*), where *m* (±1/2) and *M* (0, ±1) are magnetic quantum numbers for the electron and ^14^N nucleus (99.6% abundance) and angular-dependent *a* is the hf interaction. This gives rise to the three allowed transitions (Δ*m*= ±1, Δ*M* = 0), a triplet shown in (figure 9(B)), but for ^15^N, having nuclear spin 1/2, the spectrum consists of just two lines (a doublet). If the nitroxide spin label is free (not interacted with a protein) and tumbles fast enough, the hf anisotropy averages out and the triplet of narrow lines having almost equal amplitudes is observed. The lines are equidistant, split by the isotropic part *a*_0_ of the hf interaction. In the case of a nitroxide probe in a viscous or anisotropic media or for a nitroxide spin label covalently attached to a cysteine residue in a protein, the intensity and the width of the ESR lines depend on the intrinsic mobility of the spin label moiety and the dynamics of the protein region in the vicinity of the spin label. The spectral shape is then determined by the viscosity of the medium, local and microscopic ordering, rotational diffusion anisotropy, and macromolecule (e.g. protein) dynamics; with some or all of these parameters extracted in the data analysis.

Consequently, if the region of the spin-labeled cysteine residue is conformationally restricted and its mobility is in the range of slow dynamics, the CW ESR spectral lines are broad and of low intensity; on the contrary, if the spin label is attached to a cysteine residue in a protein region having fast conformational dynamics, the lines in the CW ESR spectrum are narrower and more intense (figures 9(C) and (D)). Still, they will be broader and of lower intensity than the lines of a free-tumbling unbound spin label in similar concentration. A qualitative analysis of such narrow-line CW ESR spectra can be done simply by comparing line heights to infer using simple formulae spin-label rotational correlation time *τ*_c_ (reflects the rotational diffusion rate *R* of the spin label), being typically in the picoseconds to nanoseconds range, depending on the spin label environment and motion anisotropy [35, 36]. A more detailed analysis of the nitroxide spectra with complex slow-motion lineshapes can be performed by using computer programs. The software programs, such as NLLS [37] or EasySpin [38], are extremely useful for comprehensive analyses of CW ESR spectra, yielding the components of rotational diffusion tensor and microscopic ordering parameters. All in all, CW ESR is a powerful technique to study local protein conformational dynamics and elucidate the effect of ligand or protein binding on the conformations of the studied spin-labeled protein.

### 3.3. CW ESR instrumentation

**Figure 10.**
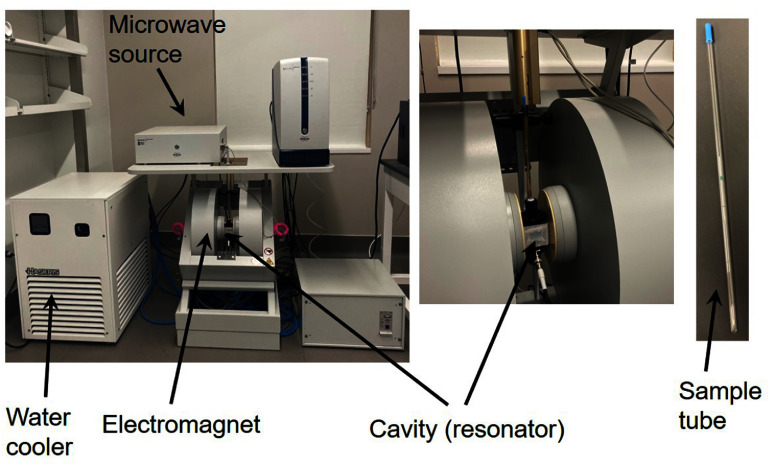
A typical X-band CW ESR spectrometer exemplified with the EMX Micro^TM^ compact type ESR spectrometer (Bruker BioSpin, Corp). The water chiller, the resistive electromagnet with its power supply on the right, the cavity (resonator) where the sample is placed, and the MW bridge (marked as ‘source’) are indicated. The signal channel and the field controller are inside the enclosure to the right of the bridge. A standard, 4 mm OD, ESR sample tube housing a 50 *µ*l microcapillary glass pipette (e.g. Kimble, green ring) containing an aqueous sample is shown on the right.

**Figure A2.**
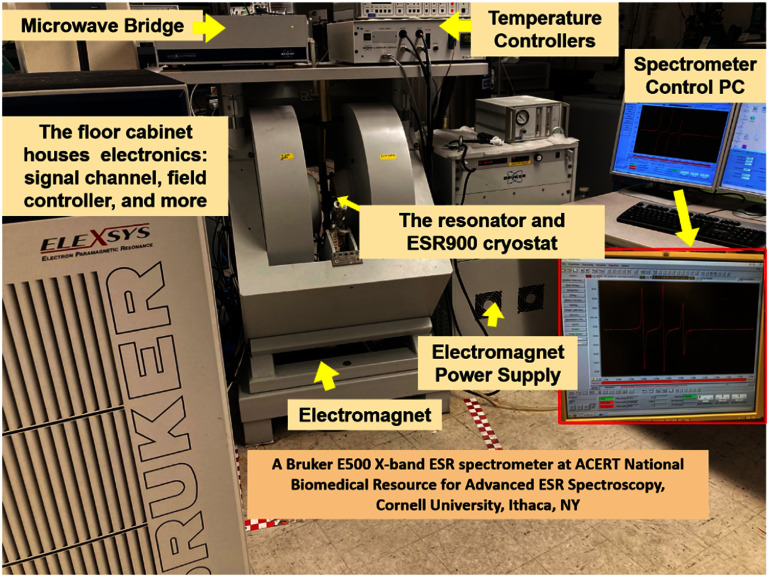
A high-end X-band CW ESR spectrometer is exemplified with a Bruker X-band ElexSys® E500 spectrometer deployed at ACERT ESR Resource. Its main components are arranged around a floor-standing resistive electromagnet with its power supply and (hidden) recirculating water-cooling unit. The microwave bridge and sample temperature controllers sit on the magnet top platform. The resonator in the magnet pole space is equipped with a cryostat. A large cabinet on the left houses all major electronics, such as spectrometer control, signal channel, field controller, and their respective power supplies and cabling. A spectrometer control PC monitor is visible, and the screen image taken with a recorded spectrum is overlapped with the photo and emphasized with a red border.

Most of the CW ESR experiments on spin-labeled proteins, and spin-labeled membrane proteins in particular, are conducted using commercial ESR spectrometers. An ESR spectrometer is usually built around an electromagnet providing the static polarizing magnetic field. The sample is placed into a MW resonator equipped with the magnetic field modulation coils and connected via transmission line to a so called MW-bridge that contains the MW source, detector, and several other key components. Other key parts of the spectrometer include sample temperature control unit; electromagnet power supply; instrument console containing signal channel, field controller, and other essential electronics. The resonator is usually a rectangular or cylindrical cavity resonator. Modern spectrometers operate under digital control of a PC and digitize the ESR signal. The CW ESR spectra recorded with a Bruker® ESR spectrometer use data acquisition server controlled by software, such as Bruker Xepr or Xenon, running in a desktop computer connected to the acquisition server. There are also ESR spectrometers from JEOL and a few other manufacturers. By far, the CW ESR spectrometers used for biological ESR operate in X-band (3 cm) frequency range, which for a typical ∼9.4 GHz frequency corresponds to the static field of 335 mT (3350 G) for nitroxides. A compact Bruker EMXmicro^TM^ X-band ESR spectrometer is shown in figure 10, and the top-of-the-line instrument, ElexSys® E500, is depicted in the Appendix, figure A2.

Typically, majority of the CW ESR experiments are conducted in X-band (∼9.4 GHz), where most of available CW ESR spectrometers operate. Of course, the vast variety of CW ESR applications from small animal imaging to material science and pharmaceuticals use a dazzling variety of ESR instruments operating from about a hundred MHz to hundreds of GHz. Spectrometers operating in Q-band (35 GHz), W-band (95 GHz), or even higher are available commercially, being used to obtain more comprehensive information at improved spectral resolution on picoliter samples [39, 40]. Multifrequency measurements of spin-labeled proteins is a useful approach to study molecular orientation, ordering and dynamic, as well as the populations of different spin label/protein conformers contributing to the CW ESR spectrum [41, 42]. However, the complexity, cost, and availability of high-field CW ESR spectrometers, particularly of a set of spectrometers working in different frequencies, limits the multifrequency applications to a few leading ESR labs and Resource Centers.

## 4. Examples of membrane proteins studied by spin labeling and CW ESR

Here, we will show two examples of protein conformational studies using nitroxide spin labeling and CW ESR spectroscopy.

### 4.1. Conformational transitions in Tau protein upon binding to liposomes

Tau is a microtubule binding and stabilizing protein, which in free state with no binding partners is highly unstructured and dynamic [43, 44]. However, under certain conditions, this protein undergoes permanent restructuring forming insoluble amyloid fibrils, a hallmark of neurodegenerative disorders, such as Alzheimer’s and other tauopathies [45]. In addition to binding microtubules, Tau associates with lipid membranes [46, 47].

**Figure 11.**
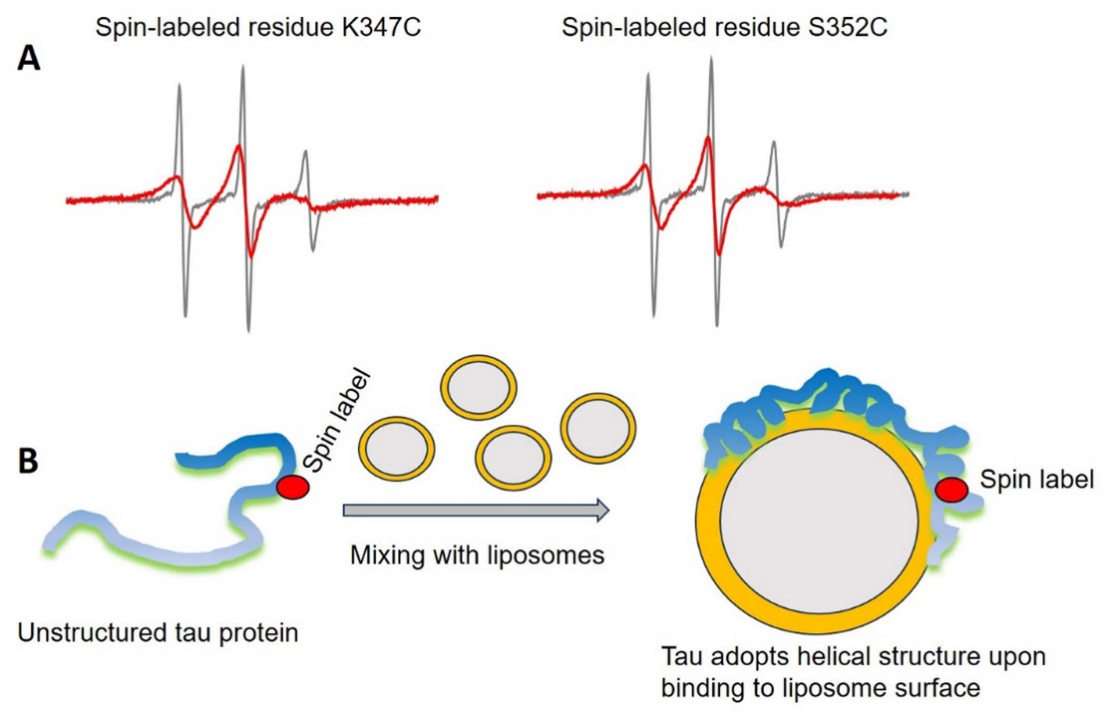
Human Tau protein was studied using CW ESR spectroscopy: (A) the CW ESR spectra for spin-labeled Tau variants with single cysteine at positions 347 (lysine was mutated to cysteine, K347C) and 352 (serine was mutated to cysteine, S352C) in buffered solution with no binding partner vs. the CW ESR spectra for the same Tau variants when bound to the surface of liposomes are shown in gray and red, respectively. Significant CW ESR spectral broadening was observed upon binding of Tau to liposomes suggesting decreased spin label mobility and protein dynamics. (B) A model of Tau’s microtubule binding domain interaction with the surface of lipid membranes—from highly unstructured in buffered solution, Tau adopts substantially helical structure on the surface of the membrane (in this case liposomes were used as membrane mimetics). The model is based on the results from [47].

The structure of Tau in solution with no binding partner and upon mixing it with liposomes was studied using CW ESR [47]. In this study, experiments on 27 spin-labeled with MTSSL cysteine substitutions in the Tau microtubule binding domain revealed a highly dynamic nature of this region in buffered solution, as the recorded CW ESR spectra for all of the spin-labeled mutants showed narrow lines (figure 11(A), gray spectra) [47]. However, the lines become significantly broader and thus less intense when the spin-labeled cysteine mutants of Tau were mixed with liposomes (figure 11(A), red spectra) [47]. This was clear indication of Tau restructuring upon the transition from its free state in solution to the membrane-bound state. After analyzing the CW ESR data for all of the 27 cysteine mutants, it was concluded that upon binding to liposomes Tau’s microtubule binding domain folds into three short alpha helices connected by much less structured, likely flexible, linkers [47]. Therefore, CW ESR was especially useful here characterizing the essential details of the structure of this protein in the context of interaction with the membrane surface. In the original study [47], no dynamics parameters for MTSSL through the corresponding Tau region were specifically followed in CW ESR spectra, but the inverse width of the central line in the CW-ESR nitroxide spectrum *(ΔH_0_^−1^*) was used to assess the position of the spin-labeled residues in respect to the membrane surface. Thus, it was found that the spin-labeled residues K347C and S352C are located on the membrane surface facing the water environment. Coarse estimation using previously simulated CW ESR spectra of nitroxide radicals [48, 49] suggests fast-motional dynamics of the spin-labeled K347C and S352C in solution with a rotational correlation time (*τ*_c_) of ⩾270 ps; whereas after binding to the liposome, the motion slows down having a *τ*_c_ of about 2–3 ns for both residues. More comprehensive data analysis beyond the scope of this work was not attempted.

### 4.2. Conformations of the influenza A M2 protein in model lipid bilayers and isolated *E. coli* membranes

**Figure 12.**
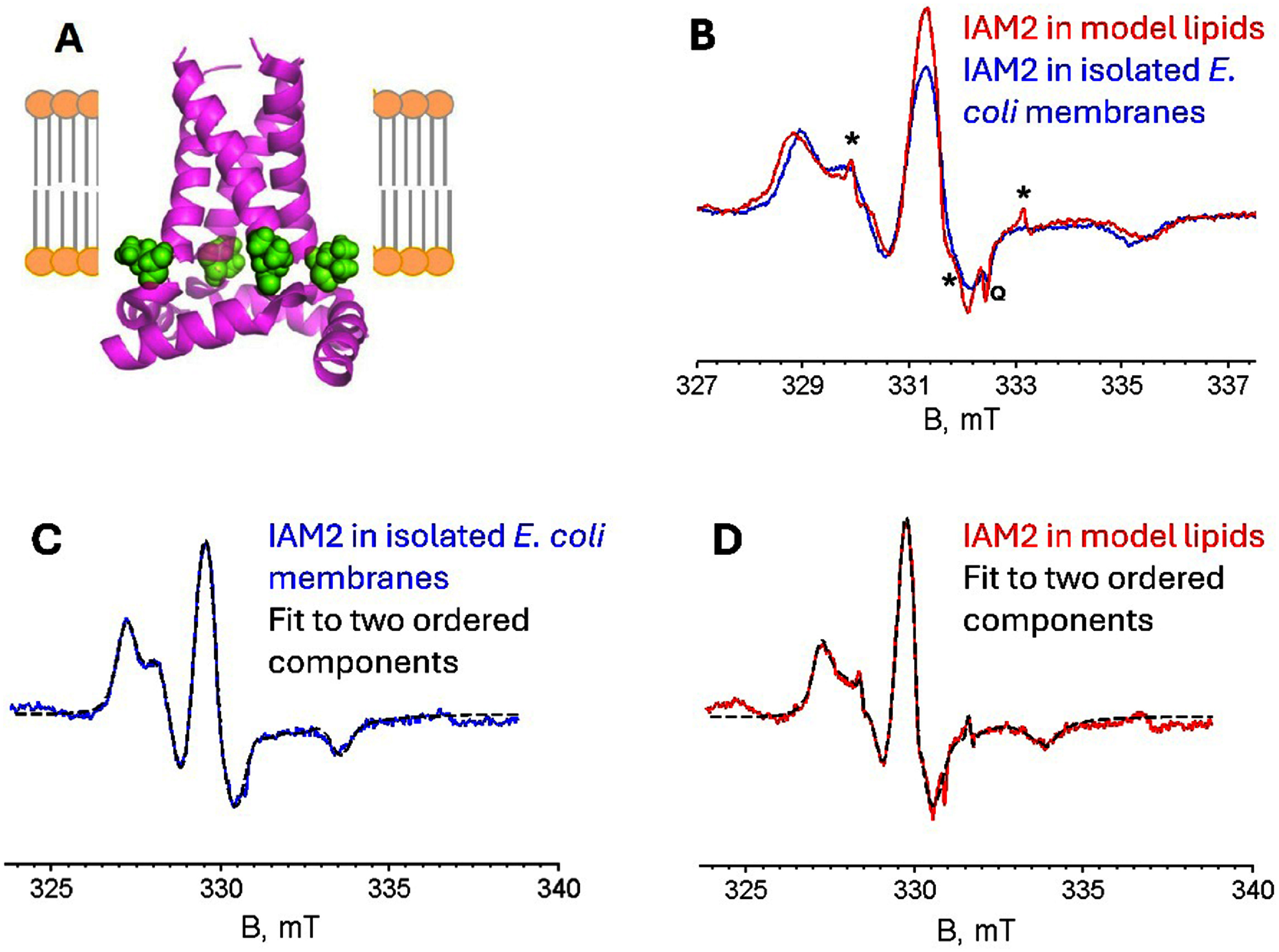
CW ESR study of influenza A M2 protein (IAM2): (A) the structure of IAM2 tetramer with residue L43 (used for spin labeling) in green are shown; (B) similar CW ESR spectra for spin-labeled at residue L43C IAM2 in model liposomes and in isolated *E. coli* membranes containing native lipids and proteins were recorded. These spectra are significantly broad suggesting substantial immobilization of the MTSSL when covalently attached to residue L43C in IAM2. The asterisks show the narrow lines of residual unreacted with the protein (free) MTSSL; Q denotes the spur from the quartz sample tube. The *B*-axis is the magnetic field in Millitesla (mT). Adapted from [32], Copyright (2024), with permission from Elsevier. (C) and (D) The CW ESR spectra of IAM2 in *E. coli* membranes (blue) and model lipids (red) fitted to a sum of two ordered components using the Multicomponent-simulation software (the envelopes of the best fits are shown in dashed black). The baseline features outside the spectral range had no role in the goodness of fit.

**Figure A3.**
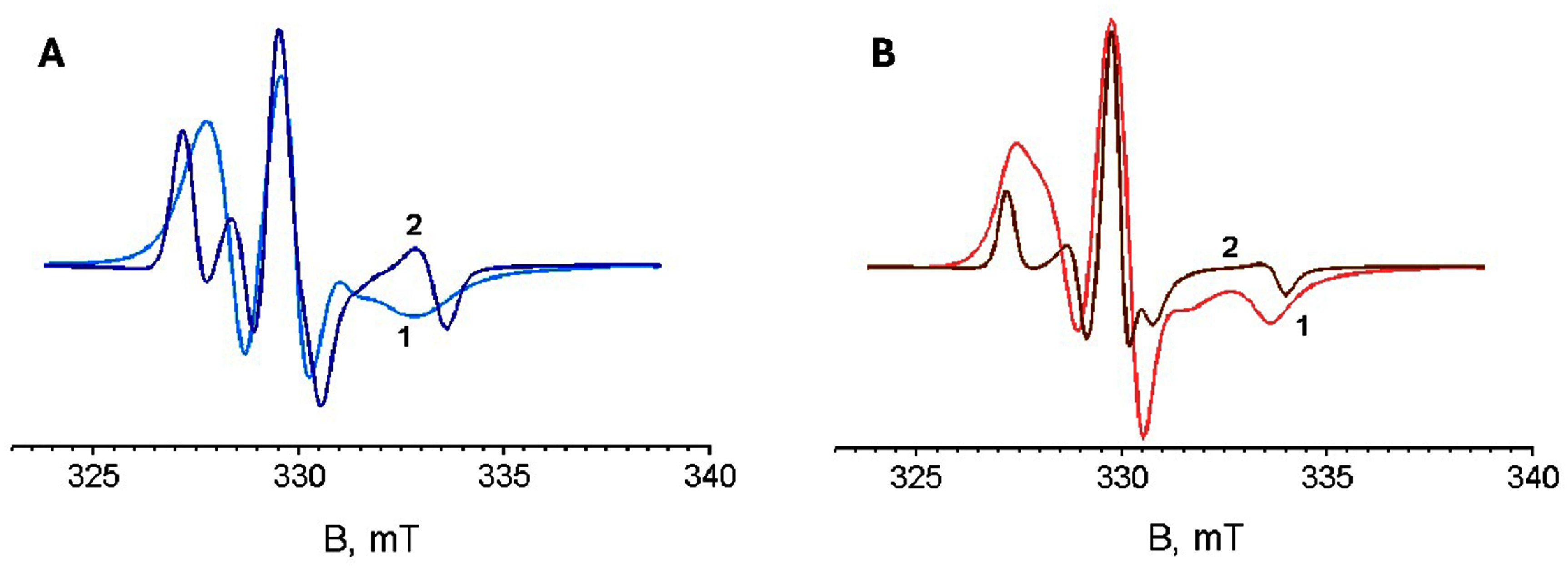
Two-component fits of the ESR spectra of the spin-labeled at position L43C IAM2 (A) in *E. coli* membrane isolate and (B) in model membranes. The ordering and dynamic parameters of each component were obtained by NLLS fitting and summarized in table A1 below.

Influenza A M2 (IAM2) is a membrane protein containing a single transmembrane helix, through which the protein oligomerizes, forming a tetrameric proton channel (figure 12(A)) [50–52]. Most of the structural and conformational studies of IAM2 have been performed on protein reconstituted in model lipid membranes with no other proteins present [25, 27, 53–55]. However, under native conditions in the membranes of infected cells, this protein shares the membrane environment with other proteins, i.e. exists under conditions of protein crowding. Recent CW ESR study was conducted on MTSSL spin-labeled mutant L43C of IAM2 in model liposomes made of synthetic lipids and also in *E. coli* membrane isolates containing native lipids and proteins (figure 12(B)) [32]. The residue L43C is located at the C-terminal exit pore of the protein channel at the membrane-solvent interface (figure 12(A)). The conformations of this region of IAM2 were found to be similar under both conditions of model lipid bilayer and isolated *E. coli* membranes, as evidenced by the high similarity of the recorded CW ESR spectra (figure 12(B)), also supported by pulse dipolar ESR. At the first glance, the CW ESR spectra of IAM2 in model and the *E. coli* membranes in figure 12(B) clearly show their slow motional nature [32], but they could also encompass significant ordering [56, 57], which is expected from MTSSL attached to a protein residue at the membrane interface. To further delve into the details of these spectra, we conducted a data analysis using a powerful fitting program, Multicomponent, developed by Altenbach and Budil [56] to fit the data for IAM2 in *E. coli* and model membranes with a sum of two ordered spectral components, since a single component failed to represent the recorded spectra. Accurate fits were generated (figures 12(C), (D), A3, and table A1) and the order parameters were obtained—high ordering was found for one of the components (cf table A1 in the appendix). This lends further support to the interpretation of the pulse ESR results in [32] indicative of two components related in concentration-dependent manner—the highly-ordered component apparently corresponds to IAM2 tetramer, whereas the less ordered one could be from a dimer or yet a form of unknown nature. This is a beautiful demonstration of the capacity of CW ESR to provide the insights that could be out of reach to other methods studying protein conformations under close to native conditions.

The technique of protein spin labeling coupled with CW ESR has been instrumental in elucidating the structure and conformational dynamics of a range of important membrane proteins, such as MsbA transporter [58], MolB_2_C_2_-A transporter [59], G-protein coupled receptors [60], ion channel [61], and many more. Extensive information can be found in excellent reviews describing the method and its applications [62, 63]. Importantly, CW ESR can help to resolve heterogeneous conformations and to report on local disorder or disorder-to-order transitions in membrane proteins, which could be otherwise difficult to achieve based on just combining current level structure prediction tools with the methods for atomic resolution structure [64].

## 5. Conclusions

Detailed understanding of the structure and conformational dynamics of membrane proteins informs us on their physiological function, but it also helps to understand in greater depth the mechanisms of malfunctions leading to disease. Biophysical techniques are instrumental in the study of membrane proteins. Typically, multiple methods are combined to assess various protein characteristics under different membrane or detergent environments, allowing for more thorough characterization of these proteins. The CW ESR spectroscopy with spin labeling is a powerful biophysical method to study membrane proteins under a wide range of lipid conditions and with few limitations to protein size or sample morphology. In this work, we visit the world of membrane proteins by sketching in a brief description of some major types. We also illustrate the general methodology of membrane proteins production in a widely used host and their purification for biophysical characterizations. The basic principles of protein labeling with nitroxide spin label are introduced and the underlying principles of protein CW ESR spectroscopy are described. Finally, we used the CW ESR studies of human Tau and IAM2 TMD to exemplify the capacity of the method to provide unique information about highly dynamic membrane-surface associated (Tau) and well-folded membrane-traversing oligomeric (IAM2) proteins. ESR is indeed particularly useful sensitive method, but as is true of any method, it has its range of limitations. For example, it would be difficult to use cysteine spin labeling to study a protein which has multiple (in some cases more than 10) endogenous cysteine residues. Mutating out these native cysteines may affect the folding, structure, and function of the protein. Therefore, alternative approaches could be to introduce unnatural amino acids or use tyrosine residues for spin labeling [65–67]. Also, the CW ESR on spin-labeled proteins has its protein and spin label concentration sensitivity limits mainly determined by the total number of spins in the sample, being also dependent on dynamics of the spin label and the protein region to which it is attached [68]. It is challenging to study proteins with a spin label in slow-motional dynamic regime at low micromolar concentrations using standard X-band CW ESR setup.

We believe that this article would be helpful for researchers planning to incorporate the technique of CW ESR into their studies of membrane proteins or hopefully in a wider range. We hope to make this method conceptually easy for trainees, motivating them to join the ranks of biophysicists.

## Data Availability

Limited new data were generated in this paper—the fitting parameters for the CW ESR spectra in figure 12 are listed in the appendix, table A1. All data that support the findings of this study are included within the article (and any supplementary files).

## Appendix

(Bottom panel) the structures of phospholipids with diverse headgroups and hydrocarbon tails: DMPC (1,2-dimyristoyl-sn-glycero-3-phosphocholine), POPC (1-palmitoyl-2-oleoyl-glycero-3-phosphocholine), DOPG (1,2-dioleoyl-sn-glycero-3-phospho-(1′-rac-glycerol) [sodium salt]), CL (1′,3′-bis[1,2-dioleoyl-sn-glycero-3-phospho]-glycerol [sodium salt], and lyso PC (1-palmitoyl-2-hydroxy-sn-glycero-3-phosphocholine) are shown. Note, glycerophospholipids typically contain 2 fatty acids (with saturated or unsaturated hydrocarbon chains) linked to the glycerol backbone via ester bonds. However, CL contains 4 hydrocarbon tails, whereas lysolipid has just one. Along with glycerophospholipids, Chol and sphingomyelin, the most abundant sphingolipid, are shown. Together, they contribute to lipid ‘rafts’ [7]. Sphingomyelin has one hydrophobic tail made of sphingosine, and the amino group of the sphingosine backbone is linked to a fatty acid via the amide bond highlighted in red. Cholesterol, abundant in central nervous system, contains a polar -OH group, sterol ring and hydrophobic tail.

CL is exclusively found in bacterial cells plasma membrane and inner mitochondrial membrane of eukaryotic cells. It is especially important for heart function, as its name may hint. Sphingomyelin is abundant in the plasma membrane and endomembranes of mammalian cells and contributes significantly to the nerve’s myelin sheath. Cholesterol plays important roles in mammalian plasma membrane, particularly in nerve cells; it modulates membrane fluidity; it is not found in bacterial membranes.

Furthermore, detailed information about membrane lipids, their distribution, and physiological role can be found in [2, 7, 69–72].

**Table A1.** The ESR data fitting parameters for panels (A) and (B) of figure A3. Diffusion and magnetic tilt angles were all less than 30° and not included; *g* and *A* tensors were common for both components. Parameters gib0 and gib2 use the standard notation for angle-dependent gaussian inhomogeneous broadening. Also, *c*_20_, *c*_22_ define ordering potential and *S*_ik_ are respective ordering parametres. Unbound spin label was fitted as a 3rd component in figure 12 D using log_10_*R* = 10 and isotropic *A* and *g* values.

	*E. coli* (panel (A))		Synthetic lipids	
Component	1, (light blue)	2, (blue)	1, (red)	2, (brown)
Gib0, G	1.3	4.0	2.0	2.7
Gib2, G	5.4	1.8	4.7	0.5
Log_10_(R_||_)	6.2	7.95	6.2	8.6
Log_10_(R_⊥_)	7.7	6.3	7.5	6.8
c_20_	0.815	3.65	1.9	5.8
c_22_	−0.28	−2.72	1.2	2.3
S_20_	0.17	0.57	0.35	0.78
S_22_	−0.08	−0.24	0.22	0.064
S_40_	0.02	0.22	0.009	0.472
S_42_	−0.015	−0.2	0.092	0.122
